# New treatment insights into pancreatic acinar cell carcinoma: case report and literature review

**DOI:** 10.3389/fonc.2023.1210064

**Published:** 2023-07-03

**Authors:** Fangrui Zhao, Dashuai Yang, Tangpeng Xu, Jiahui He, Jin Guo, Xiangpan Li

**Affiliations:** ^1^ Department of Oncology, Renmin Hospital of Wuhan University, Wuhan, Hubei, China; ^2^ Department of Hepatobiliary Surgery, Renmin Hospital of Wuhan University, Wuhan, Hubei, China

**Keywords:** pancreatic acinar cell carcinoma, chemotherapy, radiotherapy, antiangiogenic therapy, immunotherapy

## Abstract

Pancreatic acinar cell carcinoma (PACC) is a rare pancreatic malignancy with unique clinical, molecular, and morphologic features. The long-term survival of patients with PACC is substantially better than that of patients with ductal adenocarcinoma of the pancreas. Surgical resection is considered the first choice for treatment; however, there is no standard treatment option for patients with inoperable disease. The patient with metastatic PACC reported herein survived for more than 5 years with various treatments including chemotherapy, radiotherapy, antiangiogenic therapy and combined immunotherapy.

## Introduction

PACC is a rare malignancy, accounting for only 1-2% of all pancreatic malignancies. The main component is morphologically similar to alveolar cells and has the ability to synthesize exocrine enzymes. The primary site of PACC can be almost any part of the pancreas, but the head of the pancreas is the most common (1–3), with masses usually 10-11 cm in diameter (4–6).

Patients with PACC often come to the hospital with nonspecific symptoms, such as abdominal pain (60%), back pain (50%), weight loss (45%), nausea and vomiting (20%), black stools (12%), weakness, anorexia and diarrhea (8%) (7, 8). Unlike ductal adenocarcinoma, PACC rarely obstructs the bile ducts (9). Some patients may also present with lipase hypersecretion syndrome, which manifests as elevated lipase levels of more than 10,000 U or more than 10,000 U/dL (10, 11). Their levels of serum tumor markers, such as carbohydrate antigen 19-9 (CA 199) and carcinoembryonic antigen (CEA), are not consistently elevated. However, the blood levels of alpha-fetoprotein (AFP) can be elevated in younger patients (1).

The prognosis of PACC is better than that of ductal carcinoma (12, 13). Previous studies have shown that the mean overall survival time is approximately 47 months for limited disease and 14 months for metastatic disease, with 5-year survival rates ranging from 36.2% to 72.8% for surgically resected individuals (12–14).

Masses are usually detected by computed tomography (CT) and magnetic resonance imaging (MRI) and are then confirmed by fine needle aspiration (FNA) biopsy. However, MRI is superior to CT in identifying tumor margins, intratumor hemorrhage, tissue infiltration, and ductal expansion (15).

There is no clear treatment option for PACC. Some studies have shown that surgical resection significantly improves the long-term survival of patients (12). However, surgery is for only limited disease. After surgical resection, there are no standard treatment guidelines, and adjuvant therapy is individualized for most patients, with individual differences. In fact, approximately 50% of patients have metastases at the time of diagnosis (16). Metastatic sites usually include the regional lymph nodes and liver, with lung, cervical lymph node and ovarian metastases being uncommon (17). Surgery is not possible for locally advanced and metastatic disease.

A growing number of studies have demonstrated the diversity of mutated genes in PACC, with APC mutations to inactivate WNT signaling and CTNNB1 mutations to activate WNT signaling found in approximately 20% of patients with PACC (18). Even mutations in genes involved in DNA repair, such as ATM, BRCA1, BRCA2, PALB2 and MSH2, have been found in a subset of patients (19, 20), mainly manifesting as genomic instability with microvolatility of 7%-14% (18, 20). In addition, there are studies reporting significant chromosomal gains and losses in PACC patients (18, 21, 22). Performing extensive molecular analysis to identify specific genetic alterations may help to improve new therapeutic ideas.

The patients with metastatic PACC reported herein survived for more than 5 years with multiple treatment modalities applied successively, suggesting that combination therapy may be a relatively promising strategy to control tumor progression.

## Case presentation

**Figure 1:**
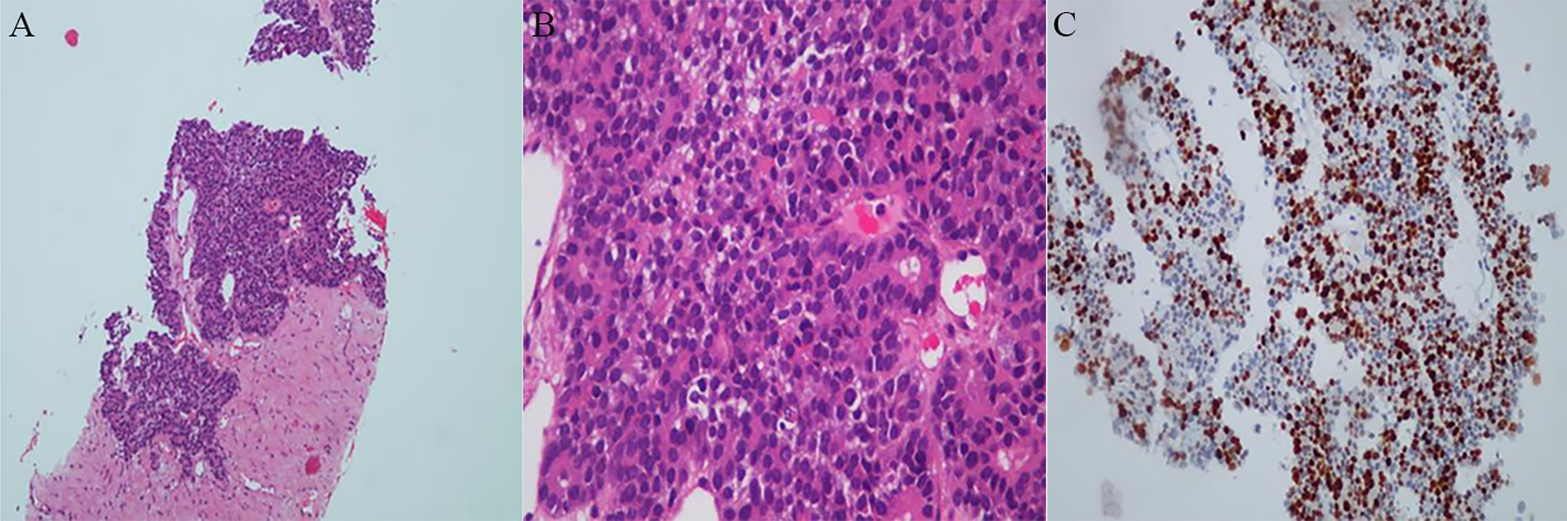
Histopathologic examinations of primary tumor. **(A)** Low magnification field of view. **(B)** High magnification field of view. A dense, nested or lamellar arrangement of tumor cells can be observed, some of which are in the form of vesicles or small glandular lumen structures. **(C)** Immunohistochemistry reaction. The results showed PCK (+), EMA (partial +), CK19 (partial +), CK7 (scattered +) Syn (scattered little +) and CgA (scattered little +).

A 44-year-old woman experienced intermittent back pain in October 2017. Positron emission tomography/computed tomography (PET/CT) demonstrated a primary tumor approximately 88*63 mm in size in the pancreatic corpus and tail; multiple lymph node metastases in the greater omentum, mesentery, hepato-renal space, and hepato-stomach space; and a metastase in the left lobe of the liver. The blood level of alpha-fetoprotein (AFP) was significantly increased, but those of carcinoembryonic antigen (CEA) and carbohydrate antigen (CA) 19-9 in this patient were normal. Ultrasound-guided biopsy of the pancreas was performed. The pathology diagnosis was pancreatic acinar cell carcinoma (PACC) (stage IV) (**Figure 1**). The results of pancreas biopsy and pathological diagnosis (November 9th, 2017) were PCK (+), EMA (partial +), CK19 (partial +), CK7 (scattered +), Syn (scattered +), and CgA (scattered +).

An adjuvant GS regimen (gemcitabine, 1000 mg/m2, Day 1 and Day 8; S-1, 40 mg/d 1–14, bid, Q21d) was initiated in December 2017. The GS regimen was stopped after two cycles because of progressive disease (PD). A chemotherapy regimen consisting of oxaliplatin plus irinotecan was used for 15 cycles from January 6th, 2018, to February 20th, 2019. The patient was reviewed periodically during treatment, and she achieved a partial response (PR) based on CT scan results until an abdominal CT scan in February showed that the numbers and sizes of primary tumor and local necrosis had increased. Therefore, treatment with single-agent albumin-bound paclitaxel was initiated. Chemotherapy was stopped after 2 cycles because of further progression of the primary tumor. Subsequently, the patient was treated with an antiangiogenic therapy, oral anlotinib (10 mg, once daily from Day 1 to 14, every 3 weeks). During anlotinib treatment, the patient underwent regular re-examination, which indicated that the patient’s condition was stable and that the primary tumor gradually decreased in size. The progression-free survival (PFS) time was 23 months.

**Figure 2:**
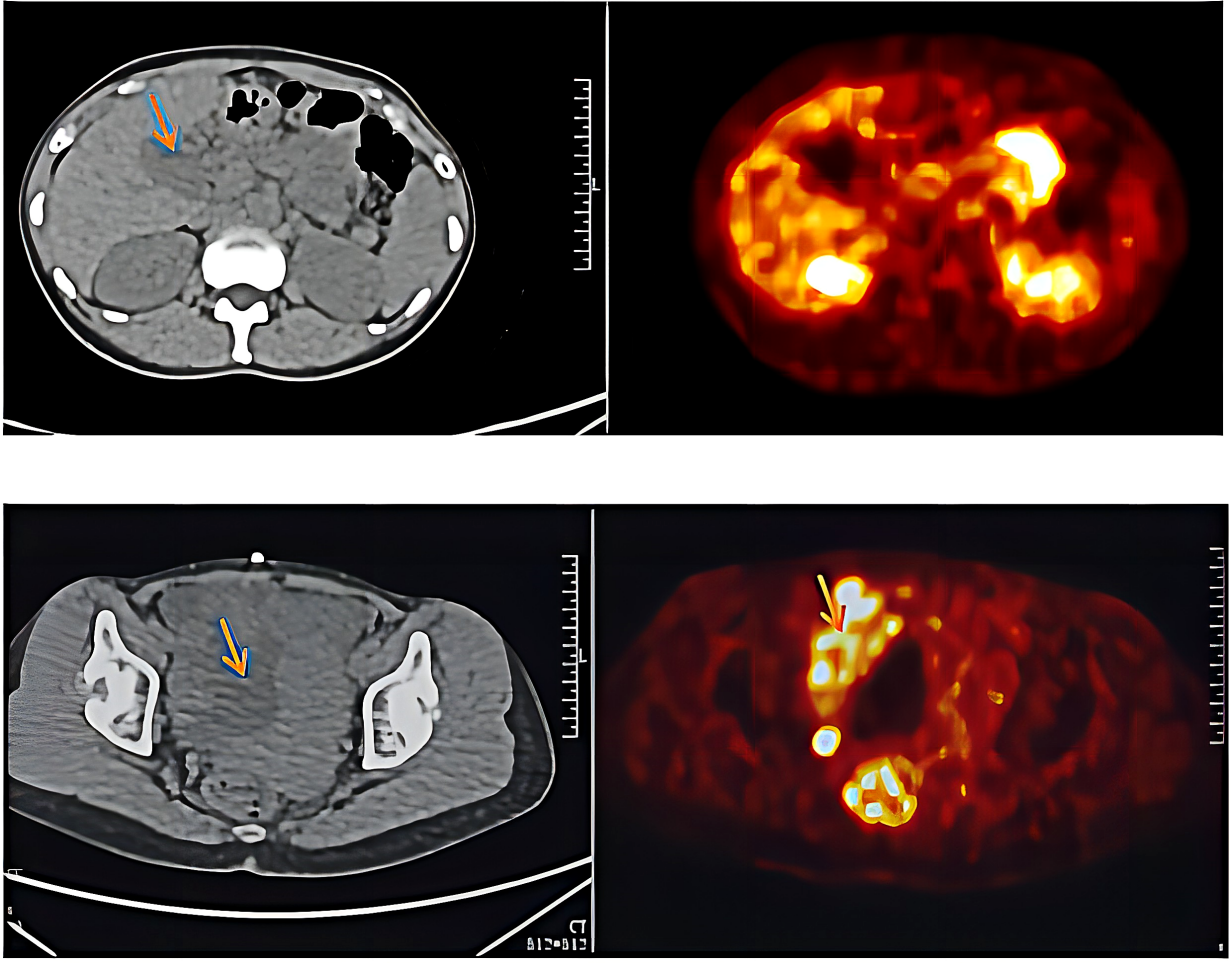
Results for PET-CT examination after chemotherapy and targeted therapy (March 10^th^, 2021).

In March 2021, the patient presented with pelvic pain without an obvious cause. The serum AFP level was increased to 109.3 mmol/L. PET/CT scan showed increased number of tumors at the primary site and detected a new pelvic metastase (**Figure 2**). Metastatic PACC was confirmed by biopsy of the pelvic metastase.

**Figure 3:**
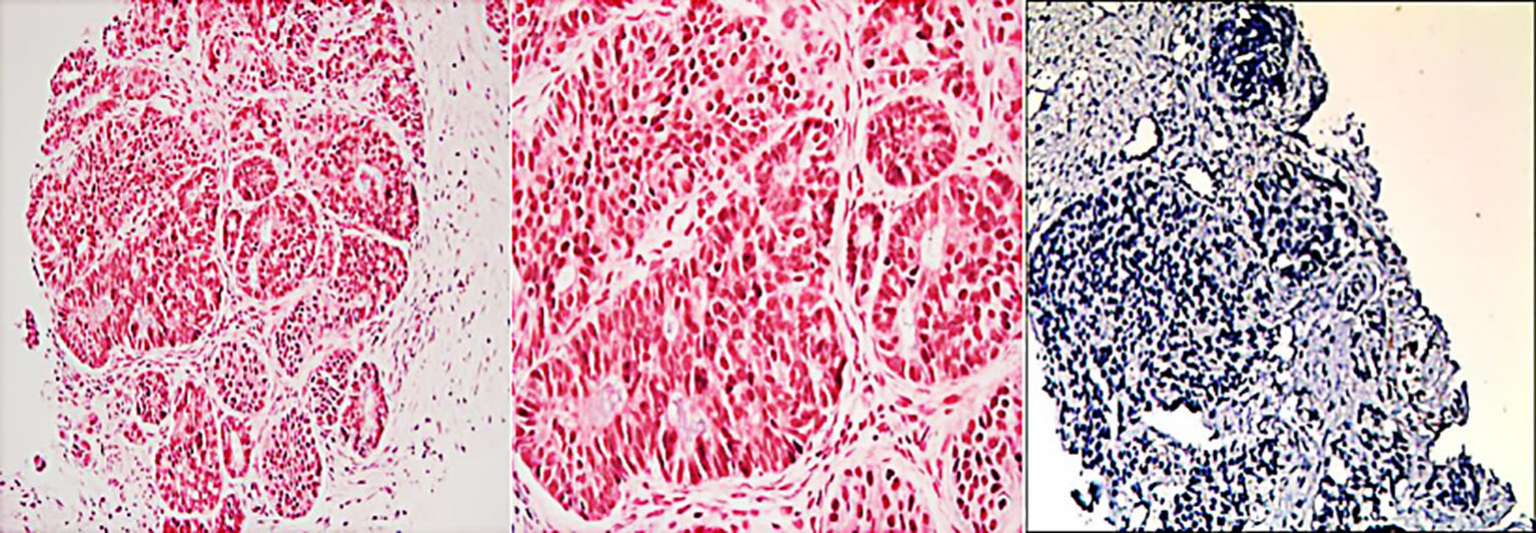
Histopathologic and immunohistochemical examinations of biopsy tissue from the pelvic metastase.

**Figure 4:**
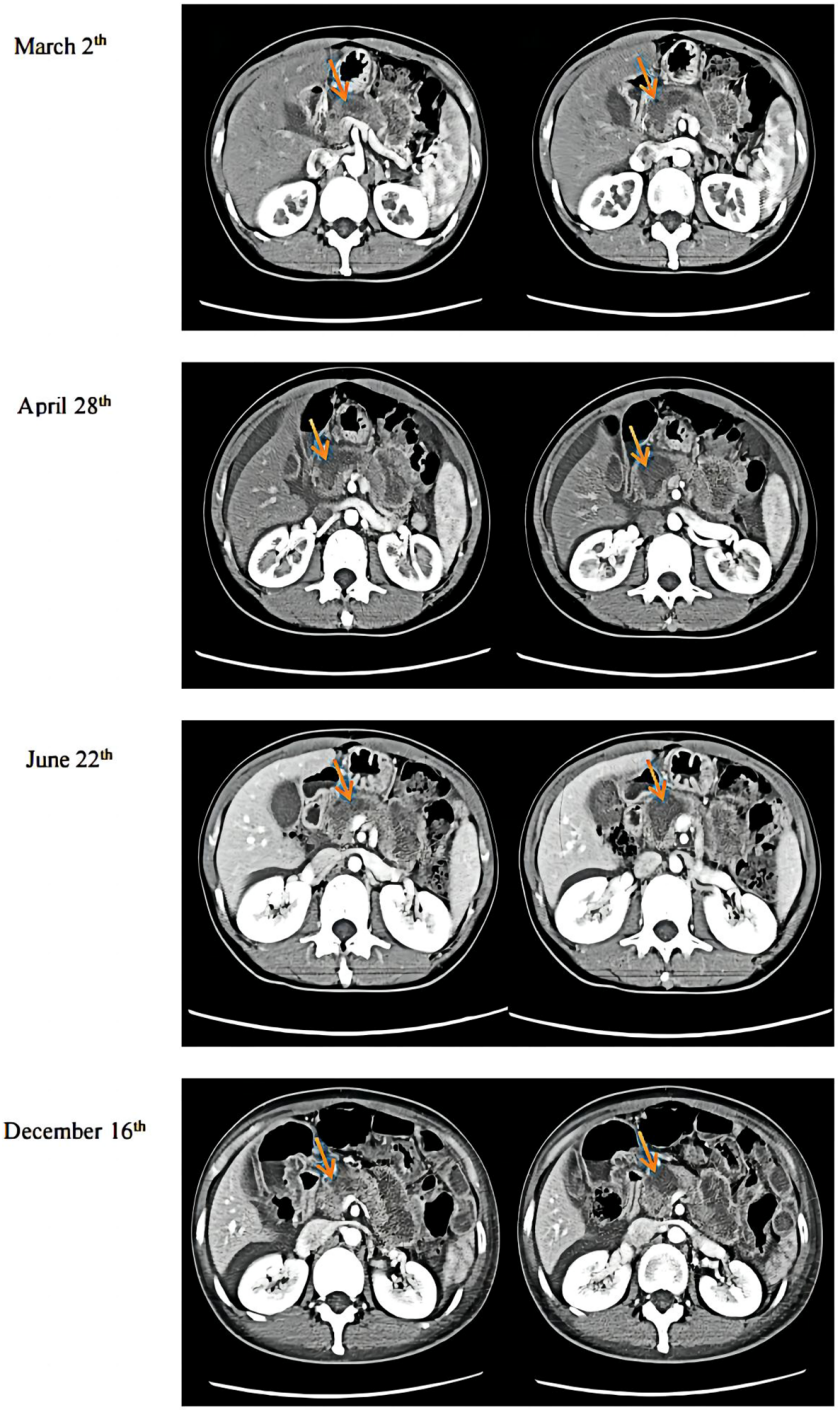
CT examination results. March 2^nd^ was during anlotinib treatment. April 28^th^ was before radiotherapy. June 22^nd^ was after immunotherapy plus radiotherapy, and December 16^th^ was during maintenance immunotherapy.

**Figure 5:**
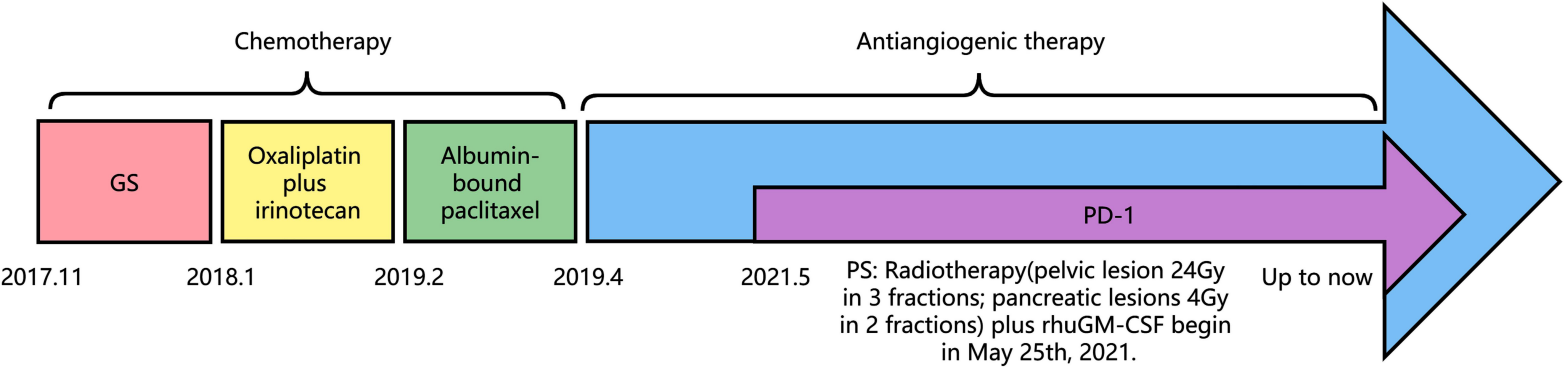
Flow chart of the treatment timeline.

Immunohistochemical results were Ki-67 (+20%), CD10 (−), CD56 (−), CgA (−), P53(+), PCK (+), SYN (+), CK7(+), CK20(−), COX-2(+), Villin (+), CDX-2(+), P40(−), Pax-8(−), SSTR2(+), and PD-1 (−) (**Figure 3**). Then, the patient was treated with radiotherapy for the pelvic metastase (24 Gy in 3 fractions) and primary tumors (4 Gy in 2 fractions). Radiotherapy began on May 25th, 2021. Meanwhile, she was treated with a PD-1 inhibitor (sintilimab, 200 mg), and recombinant human granulocyte-macrophage (rhuGM-CSF) was injected subcutaneously at a dose of 200 mg per day for 2 weeks. However, the novel anticancer oral medication had a substantially high copay per month, despite insurance coverage, making the drug unaffordable for the patient. The drug anlotinib was made accessible to the patient at no charge through support provided by the CTTQ Patient Assistance Foundation. Subsequently, the patient received anti-PD-1 therapy and antiangiogenic therapy (oral anlotinib). There was a significant decrease in the AFP level and clinical improvement to a PR for the metastatic pelvic metastase. It is important to note that all the treatments were well tolerated, with only mild toxicities. However, in August 2022, CT examination indicated disease progression with multiple metastases to the liver and bone. **Figure 4** showed the image changes of the same lesion site before and after treatment. **Figure 5** showed the treatment flow chart.

Genetic tests were performed on samples from the pelvic metastase, and the heterozygous mutation c.182A > G (p. Gln61Arg) was detected in the NRAS gene, while no mutations were detected in the BRCA gene, BRAF gene, NTRK gene, EGFR gene or TP53 gene (wild-type).

## Discussion

The prevalence of PACC has been estimated to be below 1% among all pancreatic malignancies. More than 50% of patients with PACC have metastatic disease at diagnosis. Although the reported prognosis of PACC is better than that of pancreatic ductal adenocarcinoma (23), the prognosis remains dismal. The OS time of patients with metastatic PACC is 19.6 months (24). Many studies point out surgical resection as the most effective therapeutic strategy. The survival benefit of systemic therapy is more controversial (12, 25, 26). The patient with metastatic PACC reported herein survived over 5 years through treatment with multiple therapies including radiation therapy, chemotherapy, antiangiogenic therapy, and combined immunotherapy.

PACC has unique characteristics in terms of biological behavior, imaging and prognosis relative to PDAC, such as the AFP level in some patients (27). In our case, the change in the AFP level during therapy was useful for evaluating the benefit of treatment. However, the levels of CA 19-9 and CEA, which are predictive markers for pancreatic cancer, remained normal in our case.

Surgical resection is the mainstay of treatment for PACC. It has been shown that the median survival of patients who underwent surgery was significantly better than those who did not (36 months vs 14 months) (7). Whether adjuvant therapy is recommended for patients after surgery is controversial, and most adjuvant therapy is individualised with variable and non-representative response rates. Due to the presence of genetic variants in the APC gene/β-catenin pathway in patients with PACC, chemotherapy regimens known to be active in PDAC or colorectal cancer are often used clinically (28). Combination chemotherapy regimens based on gemcitabine or fluoropyrimidine are commonly used (10, 29). No standard chemotherapy regimen has been established for patients with unresectable or recurrent PACC because PACC is a rare cancer of the pancreas, and no large-scale randomized controlled trials have yet been conducted for this disease. This group of patients usually receives neoadjuvant or palliative 5-FU chemotherapy (30, 31). Fluoropyrimidine-based combination chemotherapy can improve disease control rates (7, 10, 24, 29, 32, 33). Patients with good fitness status are treated with folinic acid/fluorouracil/oxaliplatin or folinic acid/fluorouracil/irinotecan. In contrast, patients in poor physical condition are usually treated with gemcitabine/protein-bound paclitaxel (34). Irinotecan-containing regimens are potentially beneficial regimens for unresectable or recurrent PACC (24, 26). Yoo et al. (35) confirmed that oxaliplatin-based chemotherapy had improved activity against pancreatic ACC compared to gemcitabine. Likewise, we found that the combination of oxaliplatin plus irinotecan was associated with a better response than the gemcitabine combination in our case. Compared with the benefit of gemcitabine in PDAC, the greater clinical benefit of systemic therapy observed in PACC may be explained by the greater use of combination chemotherapy regimens incorporating oxaliplatin and irinotecan over the last decade.

Myriad mutations known to have a role in tumorigenesis have been described in several PACC series (19, 20, 24, 36, 37). PACC has a genomic profile distinct from that of PDAC, with only rare mutations in TP53, KRAS, and p16, despite mutations in these genes being common in PDAC. Approximately 20% of patients with PACC have APC or CTNNB1 which could affect WNT signalling (18). Mutations in BRCA2, PALB2, ATM, BAP1, BRAF and JAK1 are likely to occur in more than a third of PACC patients (20). Genes that are involved in DNA repair, such as ATM, BRCA1, BRCA2, PALB2 and MSH2, can result in genomic instability when mutations occur (19, 20). Microsatellite instability ranges from 7% to 14% in patients with PACC (18, 20, 38). Recurrent rearrangements of BRAF and RAF1 are also present in approximately 23% of patients (37). Of these, RAF genomic alterations are observed to be mutually exclusive with altered inactivation of DNA repair genes in 45% of PACC patients (37). In addition, it has been reported that ALK mutations occur in PACC patient (39), which is quite rare, and in only 0.16% of PDAC patients (40).

There is currently no indication for tumor multigene testing for patients with advanced PACC. However, once a druggable molecular target is detected, it may improve patient survival and quality of life. Thus, a comprehensive molecular analysis was performed and the results indicated that only NRAS mutations were found, which was quite rare. The fact is that NRAS mutations occur predominantly in melanoma and the prognosis is dismal (41). Mutations in NRAS constitutively activate intracellular signaling through multiple pathways, most notably the Ras-Raf-MAPK and PI3K-Akt pathways. Activated signaling pathways can induce cell cycle dysregulation, prosurvival pathway activation, and cell proliferation (42). However, there are currently no drugs that target NRAS, but studying the signalling pathways downstream of NRAS and thus finding druggable targets has become a potential therapeutic approach (41).

Besides, inhibition of angiogenesis is an established therapeutic strategy for many solid tumors. The results of several preclinical and clinical trials have shown that antiangiogenic therapies do not improve the efficacy of pancreatic cancer treatment (43), but these trials did not involve PAAC. Anlotinib, a novel oral multitarget tyrosine kinase inhibitor, could inhibit VEGFR, PDGFR, FGFR, C-Kit, other kinases, and tumor angiogenesis- and proliferation-related signaling pathways (44). Although it has been reported in the treatment of PDAC (45), this is the first report on anlotinib treatment in advanced metastatic PACC achieving a long-term PFS time of 23 months after failure of multiline chemotherapy.

Radiotherapy (RT) is often used to “down-stage” or convert a tumor from borderline resectable to resectable and, in general, is provided as both conventional fractionation and hypofractionated stereotactic body radiotherapy (SBRT) (29). The goal of palliative RT is often to relieve pain and bleeding and/or ameliorate local obstructive symptoms in patients with nonmetastatic or metastatic disease. Besides, RT could improve survival in the metastatic disease context, which has been well established in SCLC. A systematic review revealed a modest response rate to radiotherapy in patients with localized acinar cell carcinoma of the pancreas. In several studies, a “major response” rate was observed in 25% to 35% of these patients (7, 10). However, if stable disease was included in the definition of response (i.e., the disease control rate), significantly higher response rates were seen in highly selected studies when radiotherapy was added (7, 10).

The improvement in condition of the patient after radiotherapy according to our developed protocol may be due to the following reasons. Firstly, radiotherapy can activate the immune system and trigger an antitumor immune response after cytotoxic death and immunostimulatory signal release, increasing T-cell transport to the tumor (46, 47). Although radiotherapy significantly upregulates PD-L1 expression (48), combined anti-PD-1/PD-L1 therapy counterbalances this negative effect. SBRT is more likely to induce immunogenic death in tumor cells, promote the release and presentation of tumor-associated antigens, and generate more potent systemic antitumor effects (49). Moreover, SBRT avoids lymphocytopenia, suggesting that SBRT is a better choice for combination therapy than is conventional stepwise radiotherapy.

Low-dose radiation cannot kill the tumor itself, but it is beneficial to T-cell recruitment, stromal microenvironmental regulation, and immune function promotion (50). Low-dose radiation can also improve the systemic response rates of metastatic disease treated with high-dose radiation and immunotherapy (51). In a study of a bilateral subcutaneous tumor mouse model, more than half of the mice recovered after triple treatment. This suggests that high/low-dose radiotherapy combined with anti-PD1 antibody therapy can produce a synergistic effect, and the additional low-dose radiotherapy is well tolerated by patients (52).

Immunotherapy is still at the experimental stage in the treatment of pancreatic cancer. Pembrolizumab was first included in the NCCN guidelines as a second-line treatment only for advanced solid tumors with high microsatellite instability (MSI-H) or DNA mismatch repair deficiency (dMMR). A total of 83% of pancreatic cancer patients responded to pembrolizumab treatment, with response durations ranging from 2.6 to 9.2 months (53). Tislelizumab is a humanized monoclonal antibody with high affinity and specificity for PD-1 that was specifically designed to minimize FcγR macrophage binding to eliminate antibody-dependent phagocytosis (54).

GM-CSF can enhance the effect of immunotherapy, which is closely related to its mechanism of promoting the proliferation of dendritic cells and M1-type macrophages and enhancing antigen presentation (55). Animal studies have shown that GM-CSF combined with immune checkpoint inhibitors can improve the effectiveness of PD-1/PD-L1 inhibitors by improving antigen presentation and attracting T cells to infiltrate the tumor microenvironment (56, 57). Similarly, combination therapies have been safe and effective in advanced metastatic melanoma clinical trials. In addition, some cytokines, including GM-CSF, synergize with radiotherapy (58). Abscopal responses are defined as systemic antitumor responses outside the primary radiation field. Interestingly, we found that GM-CSF combined with RT could improve the abscopal effect in preclinical data (59). A prospective study also showed that local radiotherapy combined with GM-CSF enhanced the abscopal effect (60). A phase II trial demonstrated the safety of the combination therapies. Additionally, similar clinical trials are ongoing, such as NCT04892498.

After combining high-dose radiotherapy with GM-CSF, this patient was treated with immunotherapy and achieved good disease control. Immunotherapy may benefit from combination with radiotherapy and GM-CSF due to systemic immune response activation. We should consider whether this triple therapy could provide a more diversified treatment strategy for advanced PACC and improve the poor prognosis of this disease.

Recent studies have shown that anlotinib can downregulate the expression of PD-L1 on vascular endothelial cells to alter the tumor immune microenvironment (61). NaZhou et al. (62) designed a phase IB trial to analyze the efficacy and safety of anlotinib in combination with a PD-1 inhibitor in advanced non-small cell lung cancer. The combination therapy showed favorable efficacy and manageable toxicity, especially in the 12 mg anlotinib cohort. However, the reason why anlotinib plays a vital role in treating PACC is still unclear.

## Conclusion

The rarity of PACC has led to limited recognition of the disease. Surgical resection is considered the first choice of treatment; however, there is no standard regimen for inoperable individuals. Breakthroughs in precision medicine may assist clinicians in formulating tailor-made therapies for their patients. In addition, both subtype-specific therapy and combination therapy might represent relatively promising strategies to control tumor progression.

## Author contributions

JH and JG collected data. FZ and TX wrote the manuscript. XL: Conception, organization, execution and review of manuscript. All authors contributed to the article and approved the submitted version.
